# Incremental Composition Process for the Construction of Component-Based Management Systems

**DOI:** 10.3390/s20051351

**Published:** 2020-02-29

**Authors:** Tauseef Rana, Yawar Abbas Bangash, Abdullah Baz, Toqir Ahmad Rana, Muhammad Ali Imran

**Affiliations:** 1 Department of Computer Software Engineering, MCS, National University of Sciences and Technology, Islamabad 44000, Pakistan or tauseefrana@gmail.com (T.R.); yawar@mcs.edu.pk (Y.A.B.); 2 Department of Computer Engineering, College of Computer and Information Systems, Umm Al-Qura University, Makkah P.O. Box 715, Saudi Arabia; aobaz01@uqu.edu.sa; 3 Department of Computer Science & IT, The University of Lahore, Lahore 54000, Pakistan or; 4 School of Engineering, University of Glasgow, Glasgow G12 8QQ, UK

**Keywords:** critical system, Component Based Development (CBD), composition verification, EX-MAN component model, sensors composition, incremental composition

## Abstract

Cyber-physical systems (CPS) are composed of software and hardware components. Many such systems (e.g., IoT based systems) are created by composing existing systems together. Some of these systems are of critical nature, e.g., emergency or disaster management systems. In general, component-based development (CBD) is a useful approach for constructing systems by composing pre-built and tested components. However, for critical systems, a development method must provide ways to verify the partial system at different stages of the construction process. In this paper, for system architectures, we propose two styles: rigid architecture and flexible architecture. A system architecture composed of independent components by coordinating exogenous connectors is in flexible architecture style category. For CBD of critical systems, we select EX-MAN from flexible architecture style category. Moreover, we define incremental composition mechanism for this model to construct critical systems from a set of system requirements. Incremental composition is defined to offer preservation of system behaviour and correctness of partial architecture at each incremental step. To evaluate our proposed approach, a case study of weather monitoring system (part of a disaster management) system was built using our EX-MAN tool.

## 1. Introduction

Technological advancements have made it possible to create bigger and more complex systems from the existing systems of software components and physical devices (e.g., sensors) or equipment. To keep up with the pace of this advancement and the demand for rapid application development (RAD) from the evolving market and clients, we need quicker and more economical methods for system construction. In addition, for the construction of critical system (e.g., emergency or disaster management system) [1,2], we need a safe and verifiable method. Using Internet of things (IoT)-based approaches [3,4], there are many companies (e.g., http://www.mcomo.com and https://www.inmtn.com/) offering services of critical disaster management system (DMS). In the category of natural disasters, many of the damages to human life and the physical infrastructure are caused by disasters occurring due to the changes in the weather conditions. Hardware devices to read changes in weather situations are readily available by multiple vendors. Hence, a disaster management system based on weather changes is a typical cyber-physical system (CPS) for which a safe and verifiable construction method is needed. A well-defined mechanism for the composition of existing systems into a predictable/verifiable system is the core of such a method. The essence of this composition is to provide a way to reuse existing work.

In general, the trend toward reuse in software engineering has increased the importance of composition mechanisms. Program or code reuse is one of the simplest and oldest techniques to reduce the cost by composing existing program units into larger units. The concept of ‘software reuse’ was first used in 1968 [5]. Software reuse appears at many different levels of solution development, such as code level by reusing programming language constructs (e.g., selection, sequence and looping) [6], functions/services level and data structure level. The next level is the solution domain specific and application domain specific components [7]. Software reuse, being a simple but effective technique for reducing the software development cost, appears in many forms from ad-hoc or white-box to systematic or black-box approaches [8].

In the context of the aforesaid, component-based development (CBD) can be used for the construction of management systems in a shorter time. In CBD, software components provide large-scale reuse of their intellectual property, offering reduced development and maintenance cost. Composition must be systematic or hierarchical with fixed semantics from the simplest level of program statements to the highest level of abstraction as software system, where the output of this highest level may be composed as a component to form another system. There is a close relationship between system decomposition and composition phases. The product of decomposition phase is the system architecture and the product of composition phase is the system solution. Designing a large system requires many related subsystem architectures to be composed together for system level design. Correctness of each composed subsystem architecture by a well defined composition mechanism will ensure the correctness of the composite architecture [9].

In CBD, by using a component model-based approach, solutions for a management system can be constructed from requirements directly. Each requirement builds a partial architecture; this partial architecture must be verified. With these goals in mind, first of all, we intend to find an approach providing a systemic and flexible way of composing existing components and furthermore a mechanism that allows verifying partial architecture while constructing a system. Motivated by this, from the existing approaches in CBD, we find EX-MAN component model [10] as an appropriate choice for our target goals. In this paper, we define incremental composition to construct flexible system from a set of system requirements.

In the scope of defining a safe and verifiable method for the construction of critical systems, in EX-MAN, the defined method of incremental composition has the properties of behaviour preservation and correctness-by-composition. This method can be used to construct any management system in general. However, this method with its above-mentioned properties is needed to construct critical systems. For the demonstration of the use of this method and to verify its claimed properties, we selected the DMS of wilderness weather system from Sommerville [11] with further extensions taken from Khaliq et al. [12].

The rest of the paper is organised into sux sections. To achieve our aforementioned goals, we start by investigating the architecture styles in different CBD approaches in Section 2. We select a style from this study as the best for constructing a DMS. For the selected style, we select and further extend EX-MAN component model in Section 3. Next, We define an incremental composition process for constructing flexible solutions in Section 4. For the evaluation of our approach, the details of a DMS are given in Section 5 and the implementation of this system is presented in Section 6. In Section 7, we discuss our approach and set the directions for the future work.

## 2. Flexible Software Architecture

Keeping in view the set goals in Section 1 for defining a verifiable construction method for critical systems, we study the architecture styles in different component models with respect to the units of architecture and the mechanism to compose the units. Based on our initial study, we identify two styles of architecture and find one style to be favourable for constructing systems that can also allow verifying the partial architecture during the construction process.

In general, units of architecture can be categorised into two major categories [13]: (i) components; and (ii) connectors. Components are units of behaviours as computation while connectors are descriptions of interactions between components. A component may be a physical composite or a conceptual composite of many different sub-components using different composition mechanisms. For CBD, a typical and effective composition mechanism is based on interactions between the composed components [14].

**Figure 1:**
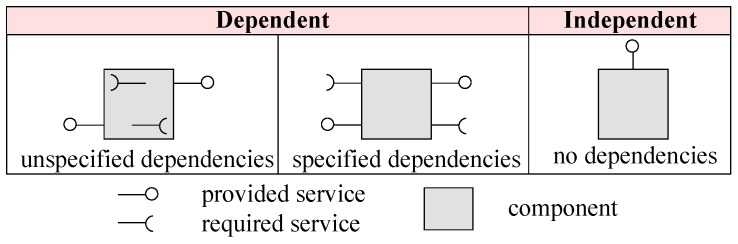
Components for system architecture.

We categorise components for system architecture into three types, as shown in Figure 1: (i) components with unspecified (hidden) dependencies [15]; (ii) components with specified dependencies; and (iii) components with no dependencies. A component is a unit of computation offering one or more provided services. A required service interface is referred to as a dependency. We categorise components from the first and second types as dependent components, and components of the third type are categorised as independent components. Dependent components depend on other components to provide their services, whereas independent components do not depend on any other component to provide their services.

To describe interactions at the architecture level, connectors are used to compose or bind components. In general, a connector can represent message passing or procedure call, event broadcasting, database queries and pipes [13].

**Figure 2:**
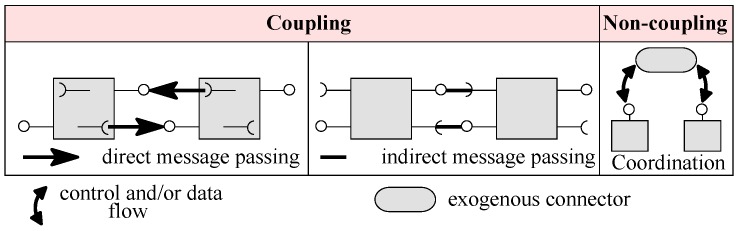
Connectors for system architecture.

In our view, connectors in a system architecture can be one of three types, as shown in Figure 2: (i) direct message passing; (ii) indirect message passing; and (iii) coordination. In direct message passing, one component’s service code has service call/request of another specific component. In indirect message passing, one component’s service code has service call/request of another component connected via a required service. In coordination, a program unit makes the service requests to the connected components without components knowing each other. We categorise the first and second types of connectors as coupling connectors, and the third type as non-coupling connectors. Coupling connectors couple two components for control flow, i.e., control initiates or flows from one component to another. On the contrary, non-coupling exogenous connectors do not couple components for control or data flows. Components are coupled with the connectors, or vice versa, for the control or data flow. There can be many different types of exogenous connectors, where each type may define a unique control and/or data flow for the composed components.

**Figure 3:**
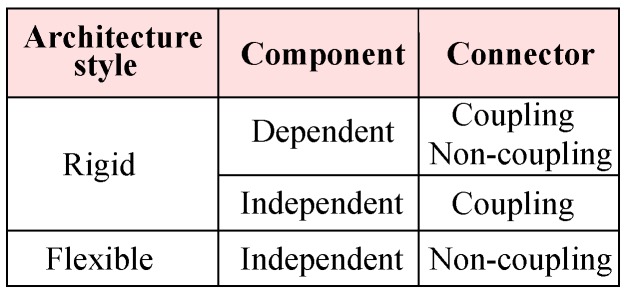
Architecture styles.

We propose two abstract architecture styles distinguishing rigid and flexible styles for software systems, as shown in Figure 3. In the rigid style, because of a dependent component or a coupling connector, it is not easy to make a change in the architecture. Contrary to the rigid style, with flexible architecture style composition, removal, replacement and reconfiguration of components without worrying about other parts of the architecture is possible [16]. In the rest of this section, we compare and contrast these styles with architecture styles from Garlan and Shaw [13].

Composition of dependent components with connectors for coupling or non-coupling defines the *rigid* system architecture. Data abstraction and object-oriented organisation style from Garlan and Shaw [13] corresponds to our category for dependent components and coupling connectors for making direct coupling between components. Event-based implicit invocation and layered systems from Garlan and Shaw [13] correspond to the same category for making indirect coupling between independent components by assuming these have no unspecified dependency. Therefore, data abstraction and object-oriented organisation, event-based implicit invocation and layered systems styles are rigid architecture styles.

Composition of independent components with connectors for non-coupling defines the *flexible* system architecture. Pipes and filters style corresponds to flexible style because filters components are independent by assuming these components have no unspecified dependency, otherwise this style corresponds to rigid style. Repositories style from Garlan and Shaw [13] corresponds to this category. Therefore repositories style is a flexible architecture style.

For CPS systems, the key issues are distribution, heterogeneity, complexity and scalability of computations devices or sensors and services. The rigid style of architecture may not be suitable as construction of such a system by using dependent components and later the dynamic configuration and maintenance of such a system would be a challenge. In contrast, the flexible style can be an easier alternative for the construction as well as the post-development maintenance of such systems. Moreover, this style can be supportive for the verification of critical systems as verifying a component’s behaviour is independent of other components in the system. Verification of a critical system is an important activity of CPS construction [17].

For an emergency and disaster management system, service-oriented architecture (SOA) is used in many approaches [18,19]. As discussed above, there are no fixed mechanisms for composition of web services. In contrast, from the same group of component models, X-MAN provides fixed connectors for composition. In view of this discussion, contrary to web services, we select X-MAN for providing a fixed set of pre-built exogenous connectors for system construction. As X-MAN has many limitations, we prefer to use extended X-MAN (EX-MAN) from Rana [10] with further modifications for critical system construction.

## 3. EX-MAN Component Model

**Figure 4:**
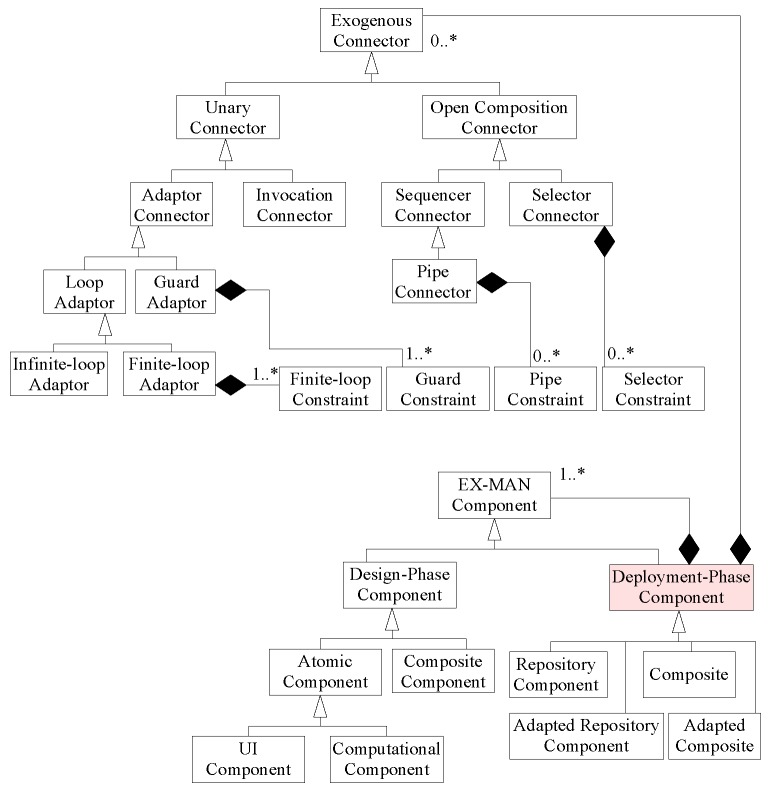
Conceptual model of EX-MAN.

**Figure A4:**
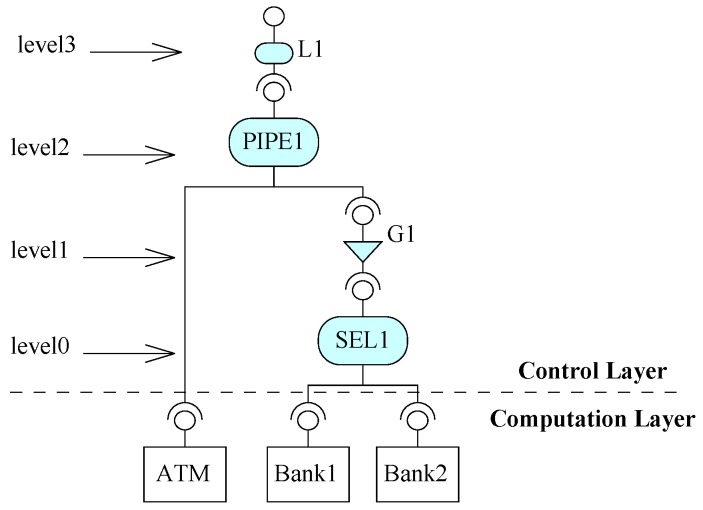
A composite of X-MAN components.

Using UML class diagram notation [20], we create the conceptual model for EX-MAN, as shown in Figure 4. The purpose of this conceptual model is to show necessary features of EX-MAN model. As shown in Figure 4, a system in EX-MAN is referred to as a deployment phase component for deployment, which is a collection of (shown by UML composition symbol) one or more components and zero or more connectors. This system can be one of four kinds, as shown in Figure 4. The system can be a collection of components and connectors, as shown in Figure A4.

**Figure 5:**
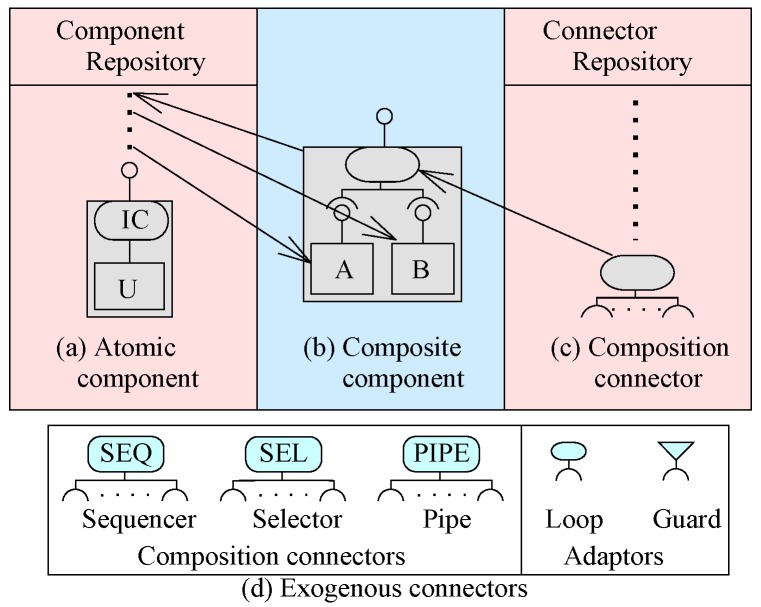
EX-MAN component model.

EX-MAN provides a fixed set of three composition and two adaptor exogenous connectors (Figure 5d). In EX-MAN, in the design phase, components for reuse are created and stored in the repository. These components are referred to as design phase components. EX-MAN defines atomic component (Figure 5a) and composition connectors (Figure 5c) to compose atomic components, in design and deployment phases. Creating a component for reuse is stored in the component repository and these components are used for system construction. An atomic component consists of an invocation connector IC to invoke a behaviour and a computation unit *U* to invoke a set of behaviours. An encapsulated atomic/composite component runs passively and it is a unit of composition. Encapsulated components do not call computation units of other components and thus have no dependencies on any other components.

Composite components are created by composing atomic components with composition connectors retrieved from respective repositories. The composite of design phase is saved into the repository of verified components for reuse, as shown in Figure 5b. The distinct feature of the exogenous composition connectors is that they encapsulate control structure to initiate control on an invocation request and coordinate the flow of data and control for composed components. Thus, encapsulated components encapsulate control, data and computation at each level of composition. In EX-MAN, the arity of exogenous composition connectors is open; this means more components can be added into a composite in multiple steps of system construction in the deployment phase. The composition connectors in EX-MAN are referred to as open connectors.

EX-MAN is a suitable candidate for flexible system architecture for three reasons: (i) decoupling the communication features as connectors from the behavioral units; (ii) ecursive connectors have a termination base case as a connector connecting only components; and (iii) all components and connectors in the system architecture are composed by a connector with one root connector.

According to the first point, composition mechanisms must be defined as a semantically independent program construct to compose software behavioural units. These independent program constructs are responsible for communication for flow of data and/or control between composed behavioural units and to the rest of the architecture. Components and connectors are reused in their own rights without any inter-dependencies.

The second point enforces the hierarchical composition in the architecture. It is this condition which makes the termination of recursive or vertical composition possible. Therefore, as per the third point, flow of control and data both have the same path and originate from the root connector.

## 4. Incremental Composition

In general, software composition means constructing bigger program units by composing smaller program units [21,22]. In this section, we define an incremental composition mechanism for EX-MAN to construct systems from a complete set of requirement specifications.

We make a number of assumptions to define incremental composition. We assume a system’s raw requirements are enumerated as a list of requirement specifications in sequence. This sequence of requirements defines the flow of actions of the system. In this paper, for incremental design from requirement, we assume any computation (e.g., start cooking, withdraw money, etc.) is a component and associations between computations (e.g., turn on the electric stove before cooking, withdraw money or inquire balance, etc.) is a connector from repository. We claim that our defined incremental composition possesses two properties: (i) behaviour preservation; and (ii) correctness-by-composition.

### 4.1. The Composition Process

**Figure 6:**
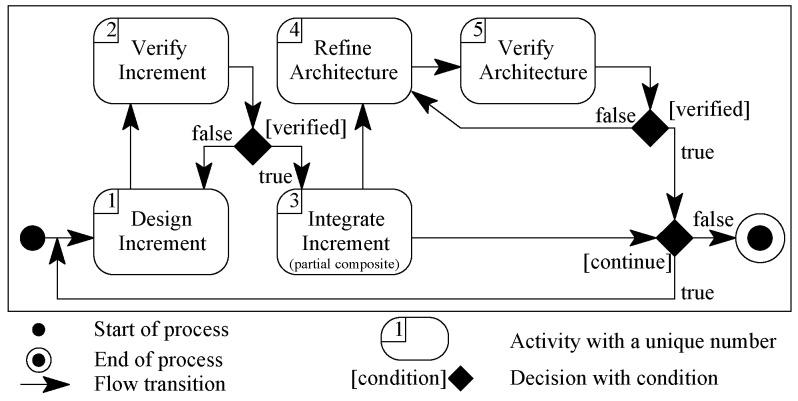
Incremental composition process.

Incremental composition is defined to address a system with a complete set of requirement specifications construction from pre-existing verified components. The composition mechanism is defined as a process of five core activities, as shown in Figure 6. The process starts with no design and the designer begins the system construction by selecting a component from a repository and the process ends with a complete system.

The first activity of the process would be to select a component from the repository for a matching computational behaviour from the requirements. This is the initial partial system to begin with. In each step of the incremental composition process, more components/connectors would be identified to be composed with the partial system. This composition will continue until the last behaviour is added from the requirements.

A pass of incremental composition process (ICP) is: (i) build increment design for a requirement as Activity 1; (ii) verify the increment design as Activity 2; (iii) integrate the increment as Activity 3 and a partial architecture from previous pass except for first requirement; (iv) in Activity 4, refine the integrated partial architecture in zero or more steps by following the refinement rules if needed; and (v) verify the integrated partial architecture in Activity 5. The composition process ends with the last pass to compose the last increment for the last requirement. For verification, each increment design is validated as per the defined component model in Section 3.

Design increment activity identify components and connectors needed from a system requirement. Integrate increment activity produces a partial architecture for the system. Partial system architecture (say X) and increment (say I) can be integrated in one of three ways:

1. Compose I with a composition connector of X.
2. Compose X with a composition connector of I.
3. Using a new composition connector, compose X and I.

A partial architecture can optionally be refined by following five rules in any order without extension and modification to the architecture:

1. Split a connector into more than one connectors of the same type.
2. Join two or more connectors of same types into one connector of the same type.
3. Remove redundant component/connector by removing the replica component/connector.
4. A selector connector can be broken into one guard per option.
5. A number of guards on the same condition with different values can be joined into a selector connector.

### 4.2. Properties of Composition

Using incremental composition, the partial system architecture preserves two properties of the system at each level of composition.

#### 4.2.1. Behaviour Preservation

We define behaviour preservation as “behavioural property of the composite architecture builds on the behavioural properties from its composed parts”. Integration of an increment *I* of some requirement with behavioural property $P_{I}$ to a partial architecture *X* from previous pass with behavioural property $P_{X}$ makes a composite architecture $X^{\prime }$ with a behavioural property $P_{I}$ and $P_{X}$. The first partial system plays the role of the base architecture.

**Figure 7:**
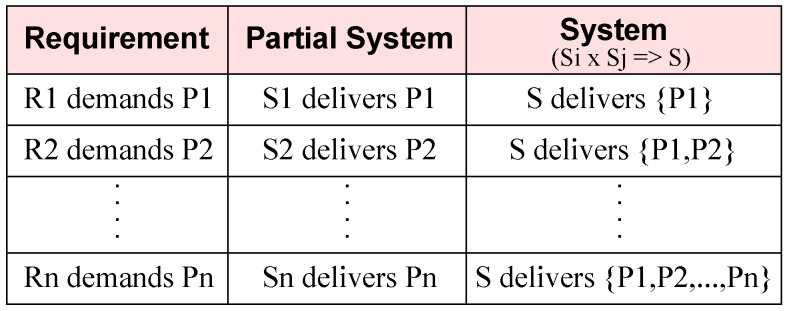
Behaviour preservation.

Each computational requirement adds some behaviour to the system architecture. In incremental composition, requirements are designed and integrated into a partial system architecture. The system’s behavioural property, after each increment, is a collection of behavioural properties of its composed partial architecture from previous pass and architecture of current increment. Hence, the final system is the collection of all the behaviours demanded in the system requirements, as shown in Figure 7. In this figure, each row represents an iteration of incrementing the partial system. The first iteration begins with no design; then, as shown by the first column, a system behaviour is identified from a requirement and a component for this identified behaviour is selected to be added in the system. The outcome of this activity is shown in the third column with the system possessing the identified behaviour in the iteration.

#### 4.2.2. Correctness-by-Composition

We adopt the definition of correctness-by-composition from Moriconi and Qian [9]: “correctness of composite architecture follows from the correctness of its parts composed architectures”. In other words, the component model of the composite is the component model of its composed parts.

The ICP verifies a requirement design as an increment *I* in Activity 2. The second execution of integration activity composes the current verified increment design of the second requirement with the verified increment design of the previous pass in Activity 5. The composite is correct by composition of two verified designs.

## 5. Disaster Management System

Disasters are either man-made (e.g., terrorist attack, cutting trees, rash or careless driving, etc.) or occur naturally (e.g., heavy rain, earthquakes, lightning, etc.) [23]. To monitor using devices/sensors [24,25], intimate to the right authorities and to take necessary actions to mitigate disasters, a computer based management system is referred to as a disaster management system (DMS). In this section, for a natural disaster management, we consider the case study of the weather station from Section 3.3 of Chapter 1 of [11] and extend it further in light of the desired features for a DMS from the works of Braune et al. [19] and Basha et al. [26].

Many natural disasters are caused by events and changes in weather situations. The damages caused by these disasters can be avoided or at least reduced with the help of a better management system. The purpose of this system is to collect data of changes in weather conditions and to raise alert alarm to the authorities to take timely actions. Manufactured by multiple vendors, there are many different kinds of devices readily available to read the changes in weather, including devices to measure wind direction and speed, air temperature, humidity in air, barometric pressure, etc. In this context, we consider a relevant case study of a wilderness weather station from Sommerville [11] and extend it further with features from Braune et al. [19], Basha et al. [26]. In this case study of a wilderness weather station (WWS), there is a weather information system (WIS) that interacts with a number of weather stations (WS) via a satellite communication system (SCS). For the extended case study of WWS, we consider a system with following requirements:

- **R1:** WS collects minimum (min), maximum (max) and average (avg) ground and air temperatures.
- **R2:** WS collects min, max and avg air pressure.
- **R3**: WS collects min, max and avg wind speed.
- **R4:** WS collects the total rain fall.
- **R5:** WS collects the wind direction.
- **R6:** Add a new sensor hygrometer to read min, max and avg humidity.
- **R7:** Keep data stored in case the data are not requested on scheduled interval because of the failure of the communication link.
- **R8:** WIS collects weather data from many WS systems installed remotely on request via a satellite communication link.

In this paper, from the extended requirements (R1–R8) for the weather station case study, initially, we identify the atomic components to be created and stored in the EX-MAN components repository for reuse in the design phase. From R1, a ground temperature sensor is connected to component GT for storing the temperature values. This component provides one service (<min,max,avg> getData()) to read the temperature data as a list of three values for maximum, minimum and average temperature values. Once read, the data storage of GT is reset; this means that the next execution of getData service provides the values computed based on data collected after previous service execution. Similarly, more components to read values from sensors are identified and created for the repository. These components are: AT (to read min, max and avg air temperature from R1), AP (to read min, max and avg air pressure from R2), WiSp (to read min, max and avg wind speed from R3), RF (to read total rain fall from R4), WD (to collect the wind direction from R5), HM (to read min, max and avg humidity from R6), DS (to store data from R7) and SC (to establish satellite communication link from R8).

For the system to fulfil Requirements R1–R8, we need many weather stations located at different areas. Hence, using the aforementioned identified atomic components from the EX-MAN repository, we need a composite component WS (weather station from R8). In the design phase of system construction, a composite component can be created by using repository components. For the construction of a composite component in the design phase or a deployable system referred to as deployment-phase component in Figure 4, our defined process of incremental composition defined in Section 4 is used.

**Figure 8:**
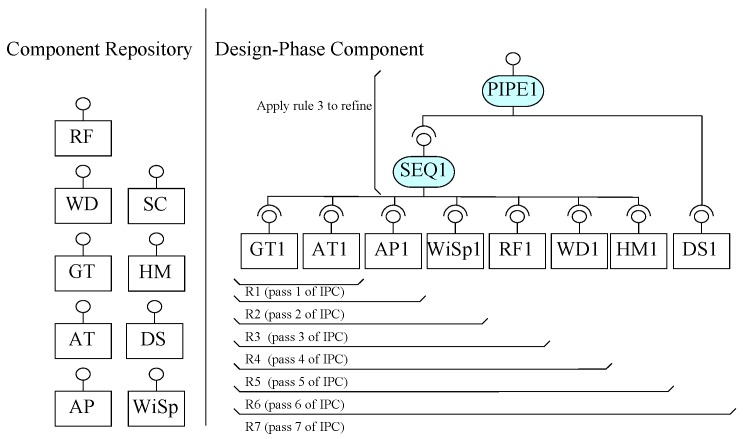
Incremental composition to compose WS.

Using ICP, we build a design phase composite component WS in seven steps, as shown in Figure 8. For the construction of this design phase component, pre-built components from the shown repository of components are selected and added into the composite in seven iterations. The connectors repository is not shown here.

In the first pass of ICP, two components, GT and AT, are identified for the construction of WS. The system needs to read respective temperature values from these two components one by one; hence, the use of a sequencer connector is identified. To design Requirement R1, Activity 1 of ICP creates a composite (shown in Figure 8 of instances of the identified components GT1 and AT1 with an instance of identified connector SEQ1). The increment is verified to produce a list of two temperature values from GT and AT, respectively. In this pass, Requirement R1 is designed. In the second pass, to design increment for R2, a component AP for air pressure is identified to be composed by a sequencer with the partial system of previous pass. Hence, an instance of the identified component AP1 is composed by SEQ1 from the partial system, as shown in Figure 8. As EX-MAN composition connectors are open in arity and the newly identified component is to be read after reading the sensors identified from R1, there is no need for any refinement in the partial architecture generated from Activity 3 of ICP. Similarly, requirements R3–R6 are designed and composed with the partial architecture in the next four passes of ICP, as shown in Figure 8. In Activity 5, for each pass after the first, the composite architecture is verified for behaviour preservation and design correctness by the defined model from Section 3.

**Figure 9:**
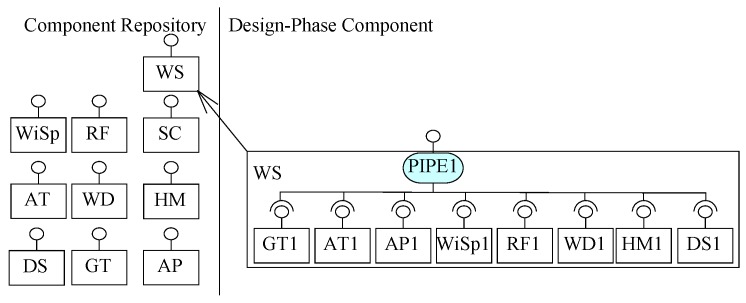
A composite component to repository.

In the next pass, to design increment for Requirement R7, DS component is identified to store the read data from different sensors designed for Requirements R1–R6. For passing data from existing composite to the DS, a PIPE connector is needed. Hence, in Activity 3 of the seventh pass of ICP, an instance of the identified component DS1 is composed with the partial architecture of the previous pass by composing through the instance of the identified connector PIPE1. In Activity 4 of this pass, SEQ1 and PIPE1 can be refined by using Rule 3 of refinement defined in Section 4.1 to be as one PIPE1 connector; this is possible because the pipe connector is a sub-type of the sequencer connector in EX-MAN, as shown in Figure 4. In Activity 5, the composite architecture is verified using the model definition from Section 3. With this pass, the weather station composite is complete and is stored in the component repository for later reuse, as shown in Figure 9. The purpose of this activity to add the composite component to the repository is to use this composite for the bigger composite construction later.

**Figure 10:**
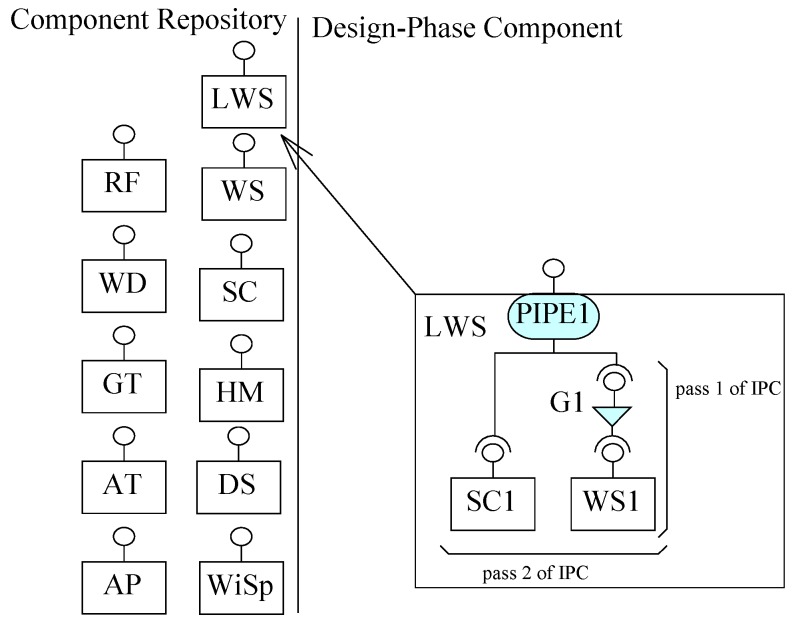
Another composite component to repository.

From Requirement R8, we identify SC and WS. The weather information system (WIS component) fetches data from more than one weather stations (WS component) by a satellite communication (SC component) link. To create a linked weather station, we create an other design phase composite (LWS) to be stored in the component repository. To get data from a WS component, the communication link must be established first. In other words, execution of the get data service from the a WS component is constrained to the satellite communication link. To gain this, in the first pass of ICP, two architecture elements a WS component WS1 and a guard connector G1 are composed to generate a partial architecture, as shown in Figure 10. In the next pass of ICP, this partial architecture is composed with SC component to create a satellite communication link by a PIPEl connector. Next the resultant architecture is stored in the component repository as a composite component. In this composite, if the connection is established by the guard connector G1, the respective service of WS1 to access data is executed. The composite component LWS is added to the repository to be used later for bigger composite construction.

**Figure 11:**
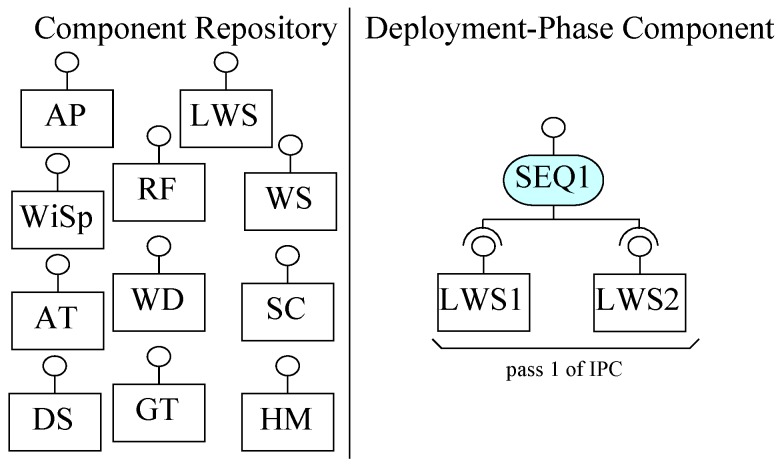
Incremental composition to construct a system.

To create the final deployable system for Requirements R1–R8, we follow ICP in the deployment phase and build the system, as shown in Figure 11. From Requirement R8, we identify sub-system weather information system (WIS) communicates with satellite linked weather stations (LWS components). For simplicity, we assume two weather stations in our design, as shown in Figure 11. In this system, the system user would request a service of the composite; this request via the sequencer connector gets data from the first linked weather station LWS1 and then data from the second linked weather station LWS2.

In the first pass ICP, two instances of LWS components (LWS1 and LWS2) are composed by SEQ1. In this design, we assume that once the system behaviour to get data from all weather stations is executed, the data from all weather stations is accessed in sequence.

WIS is the user of the system shown in Figure 11. WIS can execute the service of the composite to get data from all weather stations after some fixed time. In this architecture, EX-MAN model is used to design the weather station and WIS is a sub-system program (not developed in EX-MAN).

## 6. Implementation of Weather Station

We implemented the EX-MAN component model (shown in Figure 4) in our prototype tool called Exogenous Composition Framework (ECF). This tool is developed in Java programming language. ECF provides the semantics of EX-MAN components and connectors defined in respective classes; using these classes, a system developer can create atomic/composite encapsulated components for the component repository. Moreover, an executable system can be modeled by using the components and connectors from the respective repositories (as shown in Figure 5b).

In this section, we use ECF to construct the weather station system, as described in Section 5. The purpose of using ECF was to evaluate our proposed approach of incremental composition and the properties of composition during the process. Using ECF (in NetBeans IDE 8.0.2), initially we created nine identified (from Requirements R1–R8) atomic components shown in the component repository in Figure 8. Next, as shown in the same figure, we constructed the composite for weather station in seven ICP steps. After performing a refinement step, the composite was stored in the repository as component WS (as shown in Figure 9). Similarly, performing ICP, another composite component LWS (linked weather station) was created and stored in the component repository, as shown in Figure 10. Finally, to create the system of more than one weather stations, the composition of the deployment-phase system component is shown in Figure 11. This system is to be used by weather information sub-system.

**Figure 12:**
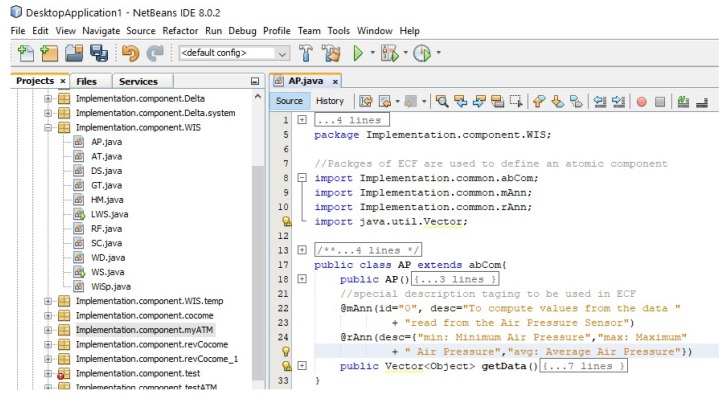
Component repository and AP Component code.

During the process of incremental composition of composite components to be stored in the repository and systems to be deployed, the intermediate composites were executed to verify the properties of the composition proposed by ICP in Section 4.2. We show the code of atomic component AP in Figure 12. The role of this component is to read air pressure values from the connected sensor and to compute the minimum, maximum and average air pressure values from these sensor read values. In WIS package, the nine atomic components and two composite components can be seen in the package in Figure 12 (left).

**Figure 13:**
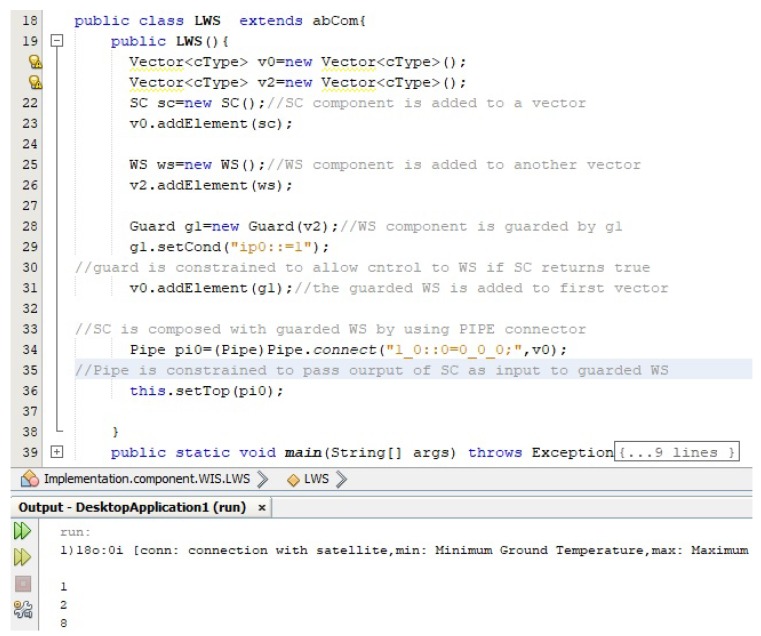
LWS composite code and component execution.

In Figure 13, the code of a composite component LWS is shown. The necessary description of the code is included as comments in the code file. In this figure, the output window shows the output of the program. The purpose of this figure is to show how the functionality of the partial system is carried out during the system construction process. With this output, the interface of the component service and the results of this service execution are printed and verified with the generated data of each component in the composite.

**Figure 14:**
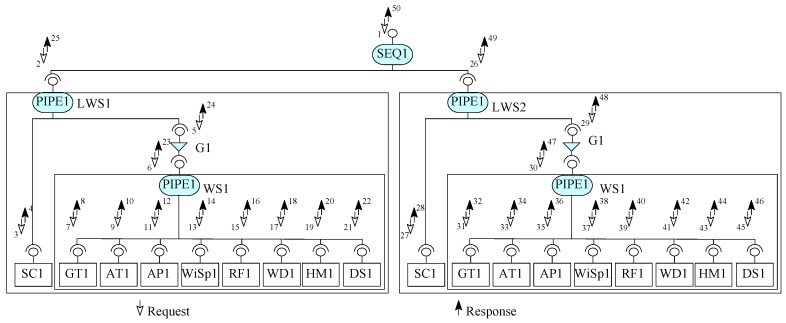
The flow of control and data in WS.

To describe the flow of control and data in the complete system in Figure 11, we show the detailed system in Figure 14. In this figure, the flow of request into a component and the flow of response out of a component is shown with the help of numeric tagging with the flow arrows. With the help of numeric tagging, we try to show the flow of requests control/data from WIS to WS, as shown in Figure 14. The shown system’s service execution begins with a request labeled “1” and ends with the response labeled “50”.

Once a request is received by the root sequencer connector SEQ1, a service request will be generated by the connector for each connected component from left to right. Hence, the next request is forwarded to LWS1 component. The root connector of LWS1 is a pipe connector. A pipe connector is a special sequencer that allows the results of one component as input data to other component in a sequence. PIPE1 of LWS1 forwards the first request to SC1 component to establish a satellite communication link with the first WS component. The result of service execution from SC1 is passed as input data for the service execution from the guard connector G1 in LWS1. G1 passes the request forward if the communication link is established. Once forwarded by G1, PIPE1 of WS initiates a request to each composed component in sequence from left to right. PIPE1 passes results of each component as input data to be stored in DS1 in WS1. In WS1, once the response is received from the last component DS1, PIPE1 generates the response for the received request. In receipt of the response from WS1, G1 generates a response, which then enables PIPE1 in LWS1 to generate a response. After receiving the response from first LWS1, SEQ1 forwards a request to LWS2. After receiving response from LWS2, SEQ1 generates response to the request generator.

## 7. Discussion and Future Work

For the development of critical system (e.g., emergency/disaster management system), the verification of the intermediate partial system architecture is an important activity. In this paper, for the unique features of EX-MAN, a process for the construction of emergency systems is proposed. In this process, the system is built step-wise and the partial architecture of the system is carefully verified during each step.

For the proposed approach, we considered an extended weather management system for its construction by using the EX-MAN component model. During the system construction, we identified a number of limitations of the proposed approach and the model used for system construction. Nevertheless, the proposed approach and the emphasis on verification and to check on process properties has introduced a new way of constructing critical systems by using EX-MAN.

The proposed process is primarily human driven; the identification of the components from the requirements set and then the composition of these components for system construction is performed by developers. Because of the experience and intuition of a developer, the attempt for a system construction by different developers would be different. However, this gives us a motivation to further study and explore the methods to support a developer as much as possible by using automated ways. In ICP, the testing of the partial architecture is also a manual process, which can also be automated to some extent with the help of a tool.

The constraint of the selected model is the passiveness of the architecture elements components and connectors. In EX-MAN system, for a component/connector to perform its tasks, the control reaches with a service request. In a cyber-physical system, the system may be comprised of many active components/sensors. For this purpose, the development approach proposed by EX-MAN should provide a means to create active components. We would like to explore and extend the model for this purpose in the future.

For demonstration and validation purposes, we use an example of a DMS; however, in general, the proposed method can be used for the construction of software-based systems and the partial systems can be verified during the construction process. For example, the CoCoMe system [27] is built using EX-MAN in [10]. The proposed method can be used to build the system with the provision to check and validate the partial systems for the behaviour preservation and correctness-by-composition. This is intended for our future work.

## Figures and Tables

**Figure A1:**
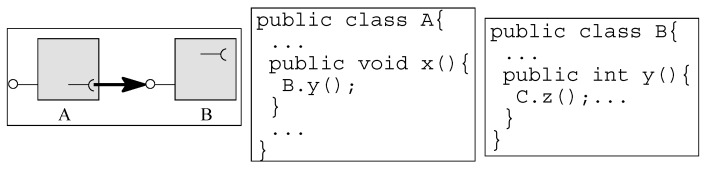
Components with unspecified dependencies.

**Figure A2:**
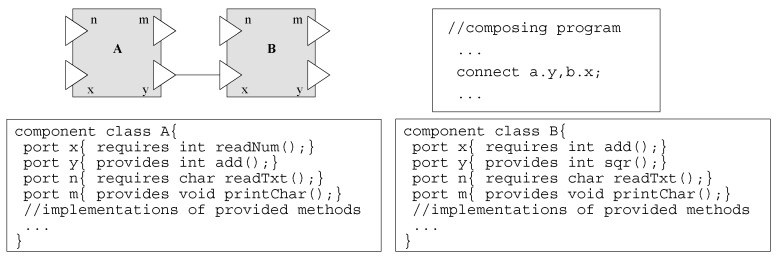
Components with specified dependencies.

**Figure A3:**
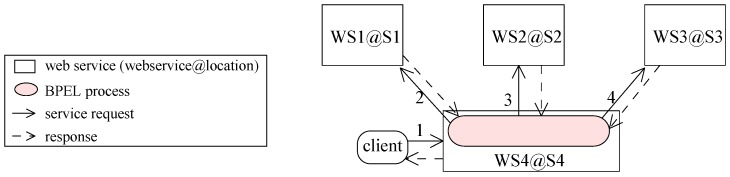
A composite of web services.

## Appendix A. Examples of System Architectures for CBD

Components with unspecified dependencies are program units depending on other program units to perform their tasks without specifying these dependencies. To use a component of this category, the user has to first find in the documentation any unspecified dependencies and then fulfil those. For example, consider a simple architecture of two components with unspecified dependencies, a class (a program unit in object-oriented programming does not specify dependencies in its interface). A calls by passing messages to class B’s functionality to perform its task. For direct message passing, A explicitly calls B with B’s reference, as shown in Figure A1. Classes A and B are coupled, control flows from A to B. Component B has an unspecified required service too, which must be fulfilled to execute the system. In this form of composition, two components are coupled at the code level. This composition is based on specific components.

Components with specified dependencies are program units with all their dependencies specified as required services along with their provided services. For example, consider an architectural unit in ADL [28] with in-ports representing required services and out-ports representing provided services. To use a component of this category, the user has to fulfil the specified dependencies. For example, consider a simple architecture of two components with specified dependencies: an architectural unit A in ArchJava [29] is connected to another architectural unit B where the connected ports must be matched and of different types, e.g., in-ports can only be connected to the matching out-ports. For indirect message passing, B calls A’s service implicitly via connected ports, as shown in Figure A2. In the perspective of control flow, architectural units A and B are also coupled. Unconnected in-ports of A and B, which are specified required services, must be fulfilled for the system to function. In this form of composition, two components are coupled at the interface level. This composition is not based on specific components but on interface. Any two components with matching interfaces can be composed.

Components with no dependencies are program units with only specified provided services. Web services [30] and components in the X-MAN component model (X-MAN) [31,32,33] do not have required interface; hence, the encapsulated component category includes X-MAN components and web services [10].

Web services and X-MAN components do not have required interface. The provided interface of X-MAN components and WSDL (web service description language [34]) of web services represent the provided service of the component. WSDL of a web service is available from a UDDI (universal description, discovery and integration) registry. Hence, in this paper, we include web services in the category of encapsulated components rather than in the category of objects as proposed in [35].

Web services are composed by orchestration [36] to produce a workflow; orchestration is a form of coordination [37,38] in which participating web services are separated from the coordination mechanism. We consider a simple online booking system Plan-a-Tour (PaT) shown in Figure A3. In this form of composition, two components are not coupled with each other. Any two components can be composed by using an independent coordinating program unit.

We assume three web services, namely WS1 to book an airline ticket, WS2 to book a hotel room and WS3 to book a taxi for airport pickup, available online on web servers S1, S2 and S3, respectively. To plan a tour (e.g., to attend a two-day international conference), a customer has to use these three web services for desired bookings for the tour. For the PaT system, using BPEL language, a workflow is created by orchestration of the three available services. The workflow is then converted into a web service WS4 by creating a WSDL for the workflow; WS4 is hosted on web server S4. A client program can call WS4 to make the three bookings in sequence.

In X-MAN, for system construction, components as well as primitive exogenous connectors are pre-built elements stored in the respective repositories. To illustrate the roles of different exogenous connectors, a simple bank system is shown in Figure A4. In this form of composition, two components are not coupled with each other. Any two components can be composed by using an exogenous connector.

The bank system in Figure A4 has one ATM (Automated Teller Machine) to serve two branches of a specific bank. The architecture of a system is a collection of components and connectors in two layers for computation and control. Due to the hierarchical nature of composition in X-MAN, exogenous connectors in the control layer can be separated into many levels. In the bank example, during the system execution, the ATM component reads an ATM card and gets the authentication from the central bank. After authentication, a composition connector PIPE1 transfers control and data to a guard connector G1. The guard connector lets the control and data flow pass thorough if the card authentication was successful. Based on the bank details, a selector connector SEL1 passes the request to one of the bank branches. After serving one customer, loop connector L1 repeats the execution to serve the next customer.
